# Effectiveness of intermittent hypoxia on muscle recovery in rats: A systematic review

**DOI:** 10.1002/ame2.70248

**Published:** 2026-07-17

**Authors:** Sebastian Philippe Hansen Quiblier, María García‐Arrabé, Diego Domínguez‐Balmaseda, Guillermo García‐Pérez‐de‐Sevilla

**Affiliations:** ^1^ Department of Physiotherapy, Faculty of Medicine, Health and Sports Universidad Europea de Madrid Madrid Spain; ^2^ Faculty of Biomedical and Health Sciences Universidad Alfonso X el Sabio (UAX) Madrid Spain; ^3^ Faculty of Medicine, Health and Sports Universidad Europea de Madrid, Escuela Universitaria Real Madrid Madrid Spain

**Keywords:** animal model, exercise, hypoxia, intermittent hypobaric hypoxia, oxidative stress, recovery

## Abstract

The objective of this review was to synthesize evidence from animal models on the effects of intermittent hypobaric hypoxia (IHH), cold exposure, and their combinations, with or without exercise, on skeletal muscle recovery after fatigue or injury. The review systematically examined controlled animal studies evaluating hypoxia‐ or cold‐based interventions for muscle recovery. The data sources PubMed, SPORTDiscus, Web of Science, and Scopus were searched up to July 2025 following PRISMA guidelines (PROSPERO CRD420251013029). Studies deemed eligible for inclusion were interventional animal studies assessing IHH, cold exposure, or combined protocols and reporting functional, physiological, or mechanistic outcomes. Risk of bias was evaluated using RoB 2. Five studies met inclusion criteria. IHH protocols simulated ~4000–4500 m for several hours per day, while cold exposure used ~4°C; some studies added low‐intensity treadmill exercise. IHH alone enhanced muscle regeneration, reduced fibrosis, and improved contractile force compared with passive recovery. Combined with aerobic exercise, IHH maintained oxidative capacity and increased PGC‐1α and VEGF expression, supporting angiogenesis. Cold exposure elevated mitochondrial complex expression but increased oxidative stress, whereas IHH improved redox balance. IHH plus light exercise also raised circulating CD34+ and endothelial progenitor cells, suggesting improved repair capacity. Overall risk of bias was rated as “some concerns,” mainly due to limited reporting of randomization. Our conclusion is that IHH shows potential to enhance muscle regeneration and redox balance in animal models. Its application in athletic recovery is promising, but standardized protocols and human trials are required before translation.

## Graphical abstract

This systematic review of animal studies evaluated intermittent hypobaric hypoxia (IHH), cold exposure, and their combinations, with or without exercise, on skeletal muscle recovery. IHH enhanced muscle regeneration, reduced fibrosis, improved contractile force, and maintained oxidative capacity, while cold exposure increased mitochondrial activity but increased oxidative stress. IHH combined with exercise promoted angiogenesis and repair capacity, highlighting its potential for improving muscle recovery.

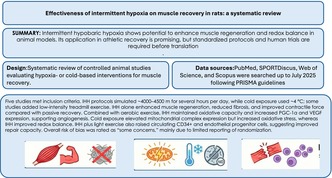

## 1 INTRODUCTION

Muscle fatigue is a main challenge in competitive sport. Beyond its immediate effect on performance, accumulated fatigue has been consistently linked to a higher risk of musculoskeletal injury. This is particularly evident in sports with congested calendars, such as professional football, where match density limits recovery opportunities. Large cohort studies have shown that injury incidence rises during periods of fixture congestion, reaching peaks of up to 27.5 injuries per 1000 match hours.^1^, ^2^, ^3^ Although the time of absence after injury may be shorter in these congested periods, the risk of sustaining an injury is greater.^4^ These effects appear to be magnified by international travel and accumulated load, while match intensity itself remains an important determinant of risk.^5^ Recent systematic reviews have confirmed that fatigue, both physical and mental, should be considered a modifiable risk factor for non‐contact injuries.^6^ Importantly, studies estimate that a considerable proportion of non‐contact muscle injuries in elite sport are fatigue‐related, underlining the clinical and practical importance of this problem.^7^, ^8^

To mitigate these effects, a variety of recovery strategies are widely used in elite sport to reduce exercise‐induced muscle damage and delayed‐onset muscle soreness (DOMS). Interventions such as cold‐water immersion, contrast water therapy, cryotherapy chambers, massage, compression garments, active recovery, and nutritional supplementation are part of daily practice in many high‐performance settings. However, the scientific evidence supporting these methods is heterogeneous. Meta‐analyses suggest that massage can reduce DOMS and improve short‐term recovery,^9^ while cryotherapy and contrast therapy show modest benefits on pain and circulating markers such as creatine kinase. Yet the magnitude of effect is often small, and methodological limitations across studies persist. No single recovery method emerges as universally effective, underscoring the need for new, more efficient, and practical interventions that could complement or improve upon current practices.

Within this search for alternatives, intermittent hypobaric hypoxia (IHH) and hypoxia‐hyperoxia have generated increasing interest. These approaches involve brief exposures to reduced oxygen availability, sometimes alternating with hyperoxic periods, designed to bring out adaptive responses. Physiological studies demonstrate that IHH can modulate motoneuronal excitability, influence autonomic regulation, and promote hematological and metabolic adaptations. Evidence from high‐altitude acclimatization indicates that chronic exposure can enhance post‐fatigue recovery, with faster restoration of quadriceps force and rate of force development compared with sea‐level or acute hypoxia conditions.^10^, ^11^ Similarly, short‐term intermittent hypobaric hypoxia protocols have been shown to evoke meaningful physiological responses, including erythropoietic adaptations, even after limited exposures.^12^ Clinical experiments in animal models further suggest that IHH may promote muscle regeneration and limit fibrosis after injury, strengthening its potential as a therapeutic strategy.^13^

Experimental work also shows that IHH can directly enhance fatigue resistance. For instance, repetitive acute IHH has been shown to increase voluntary activation and peak torque during repeated fatiguing lower limb contractions, suggesting a potential recovery‐promoting effect.^13^ Systematic reviews further emphasize the broader applications of hypoxia‐based interventions, reporting improvements in exercise tolerance, peak oxygen uptake, cognitive function, and cardiovascular parameters across varied populations, yet there is still a lack of studies that focuses directly on fatigue recovery, and until now no systematic review has directly analyzed their effectiveness for recovery from fatigue.^14^ Together, these insights reinforce the rationale for exploring IHH and related modalities within the context of muscle recovery after fatigue.

Hypoxic resistance training also provides mechanistic insights: when resistance exercise is performed under hypoxic conditions, the increased metabolic stress can potentiate hypertrophic and endurance adaptations.^15^ More recently, perceptually regulated high‐intensity intervals conducted under IHH have been shown to maintain external workload in untrained individuals, highlighting the practical feasibility of such protocols.^16^, ^17^ Importantly, clinical research suggests that acute IHH exposures of varying severities are safe and well tolerated in healthy individuals.^18^

Despite these promising findings, no human studies have yet directly evaluated hypoxia as a post‐fatigue recovery intervention in the specific context of competitive sport. Most available evidence comes either from training interventions in humans or from mechanistic studies in animal models. Moreover, systematizing studies on muscle injury recovery in humans is inherently challenging due to the substantial variability in injury characteristics (e.g., the specific muscle or muscle group affected and injury severity) and the heterogeneity of the recruited populations, which may range from professional or amateur athletes with sports‐related injuries to sedentary individuals or workers with musculoskeletal injuries. This translational gap underlines the value of preclinical studies examining the combined effects of intermittent hypobaric hypoxia, cold exposure, and exercise on skeletal muscle regeneration, oxidative stress, and mitochondrial adaptations.

The purpose of this systematic review is to synthesize experimental evidence from animal models on the effects of intermittent hypobaric hypoxia, cold exposure, and combined protocols (with or without exercise) on skeletal muscle recovery and adaptation after fatigue or injury. By clarifying the mechanistic basis of these interventions, the aim is to provide insights that can inform the design of future translational studies in humans.

## 2 METHODS

### 2.1 Study design

This systematic review was conducted in accordance with the PRISMA 2020 guidelines and was registered in PROSPERO (International prospective register of systematic reviews) on 17 March 2025, with the registration number CRD420251013029.

### 2.2 Eligibility criteria

Studies were eligible if they met the following criteria:

1. Population—animal models exposed to fatigue;
2. Interventions—any protocol of hypoxia applied as therapy for muscular post‐fatigue recovery;
3. Comparators—any type of control group;
4. Outcomes—measures of physical performance, fatigue perception, or relevant biomarkers;
5. Study design—interventional studies;
6. Language: articles published in English, French, Spanish, or Danish.

Exclusion criteria included observational designs, reviews, conference abstracts, and articles not available in full text.

### 2.3 Information sources and search strategy

Systematic searches were conducted in PubMed, SPORTDiscus, Web of Science, and Scopus, without restrictions on language or publication date. The last search was performed in July 2025. Search terms included combinations of the following keywords: intermittent hypoxia, hypobaric hypoxia, intermittent hyperoxia, muscle regeneration, skeletal muscle injury. Boolean operators (AND, OR) were applied to combine terms.

### 2.4 Study selection

All records were imported into a reference manager for duplicate removal. Then they were screened by title and abstract, and potentially relevant article were assessed full‐text by two authors.

### 2.5 Data extraction

Two research team members independently extracted data using a standardized template. Extracted information included study design, population (animal species, group size, intervention, and control groups), intervention details, and primary outcomes. Data were synthesized qualitatively and are presented in Table 1.

**TABLE 1:** Qualitative synthesis of included animal studies.

Study	Population	Intervention details	Outcomes
Santocildes et al. (2024)^21^	Population: Adult male Sprague–Dawley rats (surgically injured gastrocnemius). Groups (*n* = 6–9 per group): CTRL (injured untreated) ICE (cold exposure: 4°C, 4 h/day) IHH (intermittent hypobaric hypoxia: 4500 m, 4 h/day) ICE+IHH (combined protocol, “COHY”)	4 h/day for 9 or 21 days; hypoxia at 4500 m; cold exposure at 4°C	Day 9: IHH and ICE+IHH reduced fibrosis and improved regeneration; early contractile recovery. Day 21: sustained reduction in fibrosis, larger myofiber CSA, higher force recovery in IHH and ICE+IHH; ICE alone modest effect
Sánchez‐Nuño et al. (2024)^22^	Population: Adult male Sprague–Dawley rats (injured gastrocnemius). Groups (*n* = 4–5 per group, total 20 animals): CTRL (injured untreated) COLD (4°C, 4 h/day) HYPO (4500 m, 4 h/day) COHY (cold + hypoxia)	4 h/day for 9 days; hypoxia at 4500 m; cold exposure at 4°C	ICE ↑ ETC complex expression; IHH ↑ redox homeostasis; ICE+IHH ↑ oxidative stress & protein oxidation
Núñez‐Espinosa et al. (2015)^23^	Population: Male Sprague–Dawley rats, 350–410 g (*n* = 52 total). Groups (*n* = 8–15 depending on timepoint): UNT (untrained baseline) CTRL (eccentric exercise, passive recovery) HYP (IHH: 4000 m, 4 h/day, 21 days) EHYP (IHH + treadmill aerobic exercise, 20 min/day, 21 days)	IHH: 4 h/day, 4000 m; 21 days; exercise = treadmill 20 min/day at low intensity	IHH + exercise ↑ circulating CD34+ progenitors, EPCs, VEGF; peak at day 7; linked to muscle regeneration
Rizo‐Roca et al. (2017)^24^	Population: Adult male Sprague–Dawley rats (*n* = 32). Groups (*n* = 8 per group): CTRL (normoxia, sedentary) EX (exercise only, treadmill) HYP (IHH: 4000 m, 4 h/day, 21 days) HYP + EX (IHH + treadmill exercise)	IHH: 4 h/day, 4000 m; 21 days; treadmill exercise	HYP + EX improved muscle structural recovery, myofiber integrity, reduced damage versus controls
Rizo‐Roca et al. (2017)^25^	Population: Adult male Sprague–Dawley rats (*n* = 32). Groups (*n* = 8 per group): CTRL (normoxia, sedentary) EX (exercise only) HYP (IHH: 4000 m, 4 h/day, 21 days) HYP + EX (IHH + exercise)	IHH: 4 h/day, 4000 m; 21 days; treadmill exercise	HYP + EX enhanced hippocampal and skeletal muscle plasticity and recovery capacity; greater adaptive response than HYP or EX alone

Abbreviations: CSA, cross‐sectional area of muscle fibers; COHY, combined cold + hypoxia (ICE+IHH); CTRL, Injured untreated control (normoxia, passive recovery); EHYP, IHH + light aerobic exercise (rehabilitation treadmill); EPCs, endothelial progenitor cells; EX, exercise alone (treadmill training); ICE, cold exposure (4°C, 4 h/day); IHH, intermittent hypobaric hypoxia (≈4000–4500 m, 4 h/day); UNT, untrained baseline group (no intervention, pre‐damage); VEGF, vascular endothelial growth factor.

### 2.6 Risk of bias assessment

The risk of bias of the included studies was assessed using the RoB 2 tool for randomized trials, adapted for animal studies.^19^ Each domain (randomization process, deviations from intended interventions, missing outcome data, measurement of outcomes, and selection of reported results) was evaluated as “low risk,” “some concerns,” or “high risk.” Discrepancies were resolved by discussion between reviewers. To create a risk‐of‐bias plot, the *robvis* tool was used.^20^

## 3 RESULTS

### 3.1 Study selection

**FIGURE 1:**
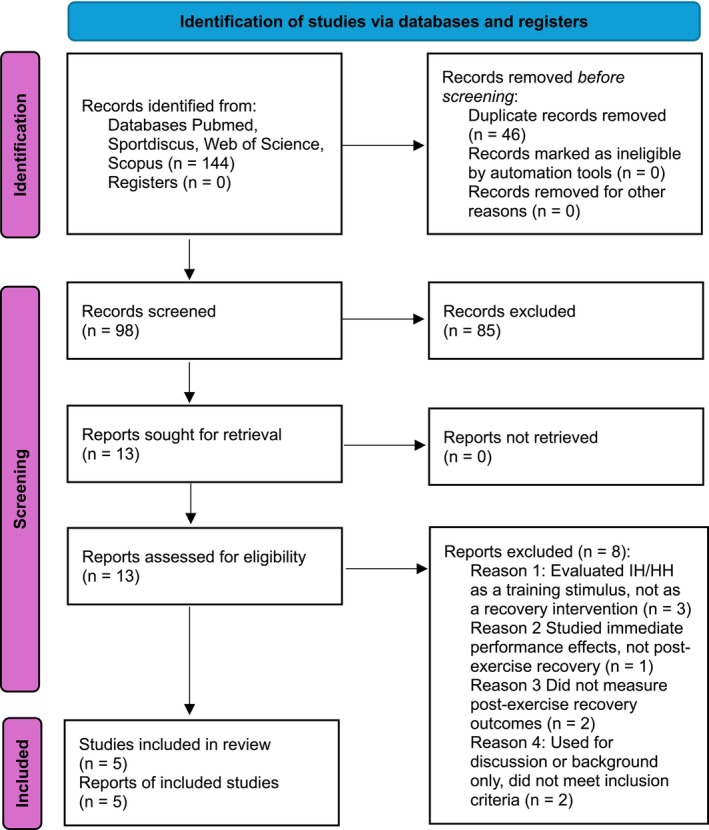
PRISMA flow diagram.

The database search across PubMed, Scopus, Web of Science, and SPORTDiscus yielded 144 records. After removal of 46 duplicates, 98 records remained for title and abstract screening. Of these, 85 were excluded as irrelevant. A total of 13 full‐text articles were assessed for eligibility, of which 8 were excluded for not meeting the inclusion criteria. Finally, five studies were included in the qualitative synthesis. The study selection process is illustrated in the PRISMA flow diagram (Figure 1).

### 3.2 Characteristics of included studies

The five studies included in this review were conducted in rat models of skeletal muscle injury or physiological adaptation and followed a controlled experimental design. Interventions included intermittent hypobaric hypoxia (IHH), cold exposure (ICE), and combinations of both, sometimes in conjunction with exercise training. Sample sizes ranged from 4 to 9 animals per group. Across the five included studies, a total of 136–150 rats animals were analyzed The duration of interventions varied between 9 and 21 days, with hypoxic exposures typically simulating altitudes of 4000–4500 m for 4 h per day, and cold exposures conducted at 4°C. The detailed characteristics of the included studies are summarized in Table 1.

### 3.3 Findings of included studies

#### 3.3.1 Fibrosis and muscle regeneration

Santocildes et al.^21^ investigated the effects of intermittent cold exposure (ICE), intermittent hypobaric hypoxia (IHH), and their combination (ICE+IHH) on muscle regeneration following a surgically induced injury in rats. After 9 days, IHH animals had already recovered contractile force and showed reduced developmental myosin expression, central nuclei, and collagen I deposition, indicating accelerated regeneration. In contrast, ICE and ICE+IHH required 21 days to reach comparable functional recovery, and ICE+IHH even blunted some of the benefits observed with IHH alone. These findings suggest that IHH is the most efficient protocol for promoting early regeneration and reducing fibrosis, while cold exposure may interfere with hypoxia‐driven adaptations.

#### 3.3.2 Mitochondrial adaptations and redox balance

Sánchez‐Nuño et al.^22^ investigated early mitochondrial and redox responses after 9 days of ICE, IHH, or their combination in a rat model of muscle injury. ICE increased the expression of electron transport chain complexes I, II, III, and V; however, this was followed by greater protein oxidation, as indicated by elevated 4‐HNE accumulation. IHH, in contrast, reduced the expression of complex III and was associated with lower oxidative damage, suggesting a more favorable redox balance. Both ICE and IHH increased eNOS expression, potentially enhancing perfusion and substrate delivery, whereas their combination (ICE+IHH) reduced ETC complex expression and blunted adaptive responses. In parallel, ICE also stimulated proteostatic responses through elevated HSP90 expression, while both ICE and ICE+IHH decreased the 19S proteasome subunit, indicating possible alterations in protein turnover. These findings highlight that IHH promotes redox homeostasis and protection against oxidative stress, whereas ICE enhances mitochondrial activity but diminishes protein oxidation, producing less consistent benefits.

#### 3.3.3 Circulating progenitor cells and angiogenic factors

Núñez‐Espinosa et al.^23^ investigated whether intermittent hypobaric hypoxia (IHH), with or without light aerobic exercise, could enhance progenitor cell mobilization during recovery from eccentric exercise‐induced muscle damage in rats. Their findings demonstrated that the combination of IHH with exercise (EHYP) significantly increased circulating CD34+/CD45– and CD34+/CD45+ progenitors, with a peak at day 7, compared with IHH alone or passive recovery. Notably, the EHYP condition also enriched the population of CD34+/DCVlow cells, considered a more primitive progenitor subset, suggesting a preferential mobilization of regenerative stem cells under this combined intervention. In contrast, IHH alone mainly increased bone marrow side population cells early (day 1), but this effect was not sustained over time. These results support the concept that exercise potentiates the pro‐regenerative effects of hypoxia by amplifying progenitor mobilization, thereby offering a mechanistic explanation for the observed acceleration of skeletal muscle repair under EHYP conditions.

#### 3.3.4 Oxidative stress and antioxidant defense

Rizo‐Roca et al.^24^ investigated the effects of intermittent hypobaric hypoxia (IHH), aerobic exercise, and their combination on recovery following eccentric exercise‐induced muscle damage in trained rats. Their findings showed that IHH combined with exercise (AHR) promoted a more oxidative muscle phenotype, evidenced by higher citrate synthase activity and increased expression of PGC‐1α, compared with passive recovery groups. In parallel, VEGF expression was elevated, reflecting enhanced angiogenesis and improved microvascular support. Although classical antioxidant enzymes such as SOD and CAT were not directly measured, the enhanced oxidative capacity and capillarization observed in the AHR group suggest a favorable redox environment that could counteract the reactive oxygen species generated after muscle damage. These results highlight the role of IHH combined with exercise in promoting mitochondrial and vascular adaptations that indirectly support antioxidant defense and muscle recovery.

#### 3.3.5 Fibrosis and muscle regeneration

In a subsequent study, Rizo‐Roca et al.^25^ demonstrated that intermittent hypobaric hypoxia combined with aerobic exercise (AHR) accelerated recovery after eccentric exercise‐induced muscle damage. After 14 days, AHR animals showed a significantly lower percentage of abnormal fibers and reduced connective tissue enlargement compared with passive normoxic (PNR) and hypoxic (PHR) recovery. These findings suggest that IHH, when combined with exercise, enhances tissue remodeling and reduces fibrosis, supporting faster functional restoration of skeletal muscle.

#### 3.3.6 Mitochondrial adaptations and redox balance

The same study reported that AHR preserved a more oxidative muscle phenotype, characterized by smaller fiber cross‐sectional area, higher citrate synthase activity, and increased PGC‐1α expression relative to PNR and PHR groups. Importantly, while passive IHH alone failed to maintain oxidative capacity, its combination with exercise reversed the decline in mitochondrial markers observed after muscle damage. This highlights the synergistic role of hypoxia and aerobic exercise in reinforcing mitochondrial biogenesis and redox balance during recovery.

#### 3.3.7 Circulating progenitor cells and angiogenic factors

Rizo‐Roca et al.^25^ also observed that AHR restored capillary density and capillary‐to‐fiber ratio to levels comparable with control animals. VEGF expression was significantly elevated in both PHR and AHR, but the angiogenic response was more effective when exercise was combined with hypoxia. In parallel, GLUT1 was upregulated only in AHR, indicating that the combined metabolic and oxygen stress more robustly activated HIF‐dependent pathways. These results suggest that IHH plus aerobic exercise enhances angiogenesis and improves oxygen delivery during regeneration.

### 3.4 Risk of bias

**FIGURE 2:**
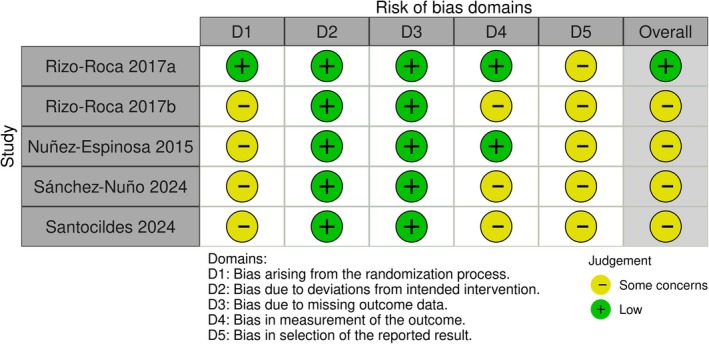
Risk of bias assessment.

Risk of bias was assessed using the RoB 2 tool.^19^ Across the five studies, the overall risk was judged as “some concerns”, mainly due to insufficient reporting of randomization and blinding procedures. Outcome measurement was generally at low risk of bias, as objective histological, biochemical, or functional measures were employed. No evidence of selective reporting was detected. Figure 2, created with the robvis tool, shows the assessment of risk‐of‐bias.

## 4 DISCUSSION

The purpose of this systematic review was to synthesize experimental evidence from animal models on the effects of IHH, with or without exercise, on skeletal muscle recovery after fatigue or injury. Across the five included studies (*n* ≈ 136–150 animals), the interventions were found to modulate several key processes involved in muscle regeneration. In particular, IHH alone or in combination with cold exposure reduced fibrosis and improved contractile recovery; IHH with exercise enhanced mitochondrial function, angiogenesis, and redox balance; and repeated exposures stimulated the mobilization of circulating progenitor cells. Collectively, these findings suggest that hypoxic protocols can accelerate muscle recovery, although the magnitude of the effect varied depending on the protocol and its combination with other stimuli.

Santocildes et al.^21^ demonstrated that daily IHH (4 h/day) reduced fibrotic tissue accumulation and promoted myofiber regeneration at both 9 and 21 days post‐injury. Importantly, IHH accelerated functional recovery compared with cold exposure, which required longer intervention to achieve comparable effects. Interestingly, the simultaneous combination of IHH with cold exposure did not provide additive benefits and in some cases blunted hypoxia‐driven adaptations, potentially due to competing or excessive physiological stress responses induced by the combined stimuli. Nevertheless, it has been suggested that the combination of IHH and cold exposure may potentiate erythropoietic responses and red blood cell‐related adaptations, which could be of interest in sports medicine contexts focused on increasing oxygen transport capacity rather than specifically enhancing skeletal muscle regeneration. These observations are consistent with preclinical models in which IHH facilitated tissue remodeling and limited fibrotic scarring.^26^, ^27^, ^28^ By contrast, normoxic injured controls consistently showed delayed or incomplete regeneration, highlighting the therapeutic relevance of hypoxic stimulation.

Concerning mitochondrial mechanisms, Sánchez‐Nuño et al.^22^ reported that IHH and cold exposure elicited distinct mitochondrial responses during muscle regeneration. Cold exposure increased expression of several electron transport chain complexes and promoted mitochondrial adaptation, although it was also associated with greater accumulation of oxidized proteins. In contrast, the combined protocol (IHH + ICE) reduced the expression of complex III, a major source of reactive oxygen species production, and prevented excessive protein oxidation, suggesting a more favorable redox profile. Notably, the simultaneous combination of cold exposure and IHH (COHY) resulted in reduced ETC complex expression, lower eNOS responses, and enhanced protein oxidation, indicating impaired mitochondrial adaptation and diminished recovery‐related responses. The authors hypothesized that the simultaneous exposure to cold and hypoxia may induce excessive catabolic and metabolic stress by increasing energetic demands while limiting oxygen availability, thereby attenuating the beneficial adaptations observed with each stimulus separately. Prior studies have similarly shown that IHH enhances mitochondrial efficiency and redox regulation,^29^, ^30^ but the lack of synergy in the combined group underlines the need to optimize multi‐modal protocols to avoid excessive oxidative stress.

Importantly, the impact of IHH is not limited to mitochondrial regulation but also extends to systemic adaptations related to tissue repair. In this regard, Núñez‐Espinoza et al.^23^ demonstrated that IHH combined with light aerobic exercise significantly increased circulating progenitor cell subsets, including CD34+ progenitors and endothelial progenitor cells, with a peak at day 7 of recovery. VEGF levels were also elevated, supporting angiogenic responses. In contrast, IHH alone induced only transient mobilization of bone marrow progenitor cells, which was not sustained over time. These results suggest that exercise potentiates the pro‐regenerative effects of hypoxia by amplifying stem and progenitor cell mobilization. Mechanistically, these adaptations are in line with hypoxia‐inducible factor (HIF)‐mediated pathways described in rodent and human studies, which link IHH to EPC recruitment and angiogenesis.^9^, ^31^ Together, these results suggest that progenitor cell mobilization may represent one of the most translationally relevant mechanisms by which hypoxia promotes muscle recovery.

Adding to this integrated view, Rizo‐Roca et al.^24^ reported that IHH combined with aerobic treadmill exercise promoted a more oxidative muscle phenotype, with higher citrate synthase activity, increased PGC‐1α expression, and enhanced VEGF levels, suggesting improved mitochondrial function and angiogenic support during recovery. These adaptations are consistent with prior models of hypoxic training, where repeated exposures elevated enzymatic and non‐enzymatic antioxidant defenses.^32^, ^33^ More recently, recovery in normobaric hypoxia has been reported to influence redox status and training adaptation,^34^ supporting the notion that controlled hypoxic stress can provide protective effects. Interestingly, IHH alone produced smaller changes, implying that multimodal approaches such as IHH combined with exercise may be necessary to optimize redox balance.

In a complementary study, Rizo‐Roca et al.^25^ found that IHH plus aerobic treadmill exercise reduced abnormal fibers and fibrosis while preserving oxidative capacity and promoting angiogenesis. Although epigenetic markers were not directly assessed in these experiments, prior evidence indicates that hypoxic exercise can influence transcriptional and epigenetic regulation in skeletal muscle. For instance, resistance training under hypoxic conditions has been shown to reverse muscle atrophy through increased acetylation of FoxO1,^35^ while in vitro studies using C2C12 myoblasts demonstrated that severe hypoxia (1% O_2_) upregulates HDAC9, thereby suppressing myogenic differentiation via reduced autophagy gene expression.^36^ Taken together, these findings suggest that IHH protocols may not only support structural and metabolic recovery but could also induce epigenetic modifications that favor long‐term muscle regeneration.

### 4.1 Limitations

Several limitations should be acknowledged. Regarding generalizability, all included studies were performed in male rodents, which may not fully replicate the complexity of human muscle physiology and limits the extrapolation of findings across sexes. Sample sizes were modest (typically 4–9 per group), and outcome measures were heterogeneous, ranging from histology to biochemical markers. Reviews of translational research highlight that positive preclinical findings often fail to replicate in humans due to interspecies differences.^37^, ^38^ Moreover, the hypoxic protocols differed in exposure duration, frequency, and combinations with other interventions, complicating direct comparisons across studies. Additionally, all included studies were conducted by the same research group, which may limit the external reproducibility and independence of the available evidence, despite the consistency of the findings across studies and their agreement with broader mechanistic literature. Furthermore, long‐term outcomes beyond 21 days were not assessed, leaving the persistence and clinical relevance of these adaptations uncertain. Finally, it was not possible to perform a meta‐analysis, due to the limited number of studies included.

### 4.2 Clinical implications

Despite these limitations, the findings provide mechanistic evidence that hypoxic conditioning may complement or surpass conventional recovery methods such as cryotherapy, massage, or compression garments. Systematic reviews of recovery strategies in football have highlighted the modest and inconsistent benefits of current approaches.^9^, ^39^, ^40^ By contrast, IHH targets deeper processes, including angiogenesis, mitochondrial efficiency, redox regulation, and epigenetic remodeling. These properties suggest that hypoxic protocols may have translational potential in high‐performance sport, especially when combined with light aerobic exercise. However, the clinical implementation of hypoxic conditioning remains challenging due to the difficulty in defining the optimal hypoxic dose, including exposure duration, number of sessions, severity of hypoxia, and modality of exposure. In addition, physiological responses may differ between normobaric and hypobaric hypoxia, which should be carefully considered when translating experimental findings into applied settings. Therefore, controlled human trials are required to establish the safety, efficacy, and standardization of these interventions.

### 4.3 Futures lines of research

Future research should focus on translational studies involving humans to evaluate the feasibility and effectiveness of IHH protocols in recovering from exercise‐induced muscle damage. Standardizing exposure regimens, monitoring redox and regenerative markers, and conducting controlled comparisons with existing therapies will be crucial. Lastly, mechanistic studies with athletes are necessary to determine whether the epigenetic and progenitor cell responses seen in rodents can be replicated in humans, thus bridging the gap between experimental models and clinical application.

## 5 CONCLUSION

IHH shows promise as a recovery strategy, enhancing muscle regeneration, redox balance, and repair mechanisms in animal models. These findings suggest potential benefits for athletes recovering from exercise‐induced muscle damage. However, clinical translation requires standardized protocols and controlled human trials to confirm efficacy and safety in sports settings.

## AUTHOR CONTRIBUTIONS

**Sebastian Quiblier:** Conceptualization; data curation; formal analysis; investigation; methodology; writing – original draft. **María García‐Arrabé:** Conceptualization; data curation; methodology; supervision; validation. **Diego Domínguez‐Balmaseda:** Conceptualization; data curation; formal analysis; supervision; validation. **Guillermo García‐Pérez‐de‐Sevilla:** Conceptualization; data curation; formal analysis; methodology; supervision; validation; writing – original draft; writing – review and editing.

## FUNDING INFORMATION

The authors received no funding for this work.

## CONFLICT OF INTEREST STATEMENT

The authors declare no conflicts of interests.

## ETHICS STATEMENT

None.

## PROSPERO REGISTRATION

This systematic review was registered in PROSPERO (International prospective register of systematic reviews) on 17 March 2025, with the registration number CRD420251013029.

## Data Availability

The data supporting the findings of this study are available within the article.
